# Development of a T Cell Receptor Targeting an HLA-A*0201 Restricted Epitope from the Cancer-Testis Antigen SSX2 for Adoptive Immunotherapy of Cancer

**DOI:** 10.1371/journal.pone.0093321

**Published:** 2014-03-28

**Authors:** Daniel Abate-Daga, Daniel E. Speiser, Nachimuthu Chinnasamy, Zhili Zheng, Hui Xu, Steven A. Feldman, Steven A. Rosenberg, Richard A. Morgan

**Affiliations:** 1 Surgery Branch, National Cancer Institute, National Institutes of Health, Bethesda, Maryland, United States of America; 2 Department of Oncology, Ludwig Center, University of Lausanne, Lausanne, Switzerland; University of Pittsburgh, United States of America

## Abstract

The clinical success of adoptive immunotherapy of cancer relies on the selection of target antigens that are highly expressed in tumor cells but absent in essential normal tissues. A group of genes that encode the cancer/testis or cancer germline antigens have been proposed as ideal targets for immunotherapy due to their high expression in multiple cancer types and their restricted expression in immunoprivileged normal tissues. In the present work we report the isolation and characterization of human T cell receptors (TCRs) with specificity for synovial sarcoma X breakpoint 2 (SSX2), a cancer/testis antigen expressed in melanoma, prostate cancer, lymphoma, multiple myeloma and pancreatic cancer, among other tumors. We isolated seven HLA-A2 restricted T cell receptors from natural T cell clones derived from tumor-infiltrated lymph nodes of two SSX2-seropositive melanoma patients, and selected four TCRs for cloning into retroviral vectors. Peripheral blood lymphocytes (PBL) transduced with three of four SSX2 TCRs showed SSX2_41-49_ (KASEKIFYV) peptide specific reactivity, tumor cell recognition and tetramer binding. One of these, TCR-5, exhibited tetramer binding in both CD4 and CD8 cells and was selected for further studies. Antigen-specific and HLA-A*0201-restricted interferon-γ release, cell lysis and lymphocyte proliferation was observed following culture of TCR engineered human PBL with relevant tumor cell lines. Codon optimization was found to increase TCR-5 expression in transduced T cells, and this construct has been selected for development of clinical grade viral vector producing cells. The tumor-specific pattern of expression of SSX2, along with the potent and selective activity of TCR-5, makes this TCR an attractive candidate for potential TCR gene therapy to treat multiple cancer histologies.

## Introduction

Recent advances in the fields of tumor immunology, cancer genomics and gene transfer technologies have permitted the development of therapies based on adoptive transfer of autologous tumor-reactive T cells for the treatment of human malignancies [1], [2]. Tumor-reactive T cells can be natural, as in the case of tumor infiltrating lymphocytes (TIL) purified from resected lesions and stimulated *ex vivo*, or generated from peripheral blood by introduction of genes encoding for immune receptors [3].

While the high rate (50–70%) of objective clinical responses achieved by TIL treatment in patients with advanced-stage melanoma established a solid proof of concept for the treatment of human cancers with T cells [4], [5], its widespread application is limited by the difficulty in culturing and expanding these T cells to clinically relevant numbers for every patient. As an alternative approach, the antigen specificity of readily available peripheral blood T cells can be redirected to antigens expressed in tumor cells by genetic modification. Adoptive transfer of autologous T cells engineered to express T cell receptors (TCRs) or antibody-derived chimeric immune receptors can result in a potent cellular immune response against tissues expressing the target antigens, even at low levels [3], [6]–[8]. It is therefore instrumental to carefully select antigens that are expressed in tumor cells but absent from essential normal tissues, in order to avoid undesired on-target/off-tumor toxicities.

Current efforts in identifying the optimal target antigens for adoptive immunotherapy are mainly focused on the neoantigens generated by somatic mutations present in tumors but absent in normal tissues [9], [10], on antigens expressed on dispensable normal tissues, and on a group of genes that encode for the cancer-testis (CT) antigens. The latter are defined by their pattern of expression which, in adults, is generally restricted to non-MHC expressing germ cells of testis, that thus do not present antigens to T cells, and to tumor cells of diverse origin [11]–[13]. Adoptive transfer of autologous peripheral blood lymphocytes expressing a TCR specific for a cancer-testis antigen, NY-ESO-1, mediated objective tumor regressions in patients with advanced melanoma and with synovial cell sarcoma, with no NY-ESO-1 related toxicity [14].

In order to expand the repertoire of antigens that can be targeted by this approach, therefore expanding the number of patients and tumor types that can be treated, we developed a TCR-expressing vector targeting a member of the synovial sarcoma X breakpoint family, SSX2. The synovial sarcoma X breakpoint (SSX) genes are located on the X chromosome and encode a family of ten highly homologous nuclear proteins, SSX1–10. SSX2 was originally identified as part of a genomic translocation present in synovial sarcoma [15], [16] and later found to be identical to HOM-MEL-40, an immunogenic protein known to induce spontaneous antibody responses in 10% of patients with melanoma [17].

We generated retroviral vectors encoding the TCR alpha- and beta-chains targeting the HLA-A*0201-restricted epitope SSX2_41-49_, isolated from previously described melanoma patients displaying active immune responses to SSX2 [18]. We further demonstrated that T cells engineered with these vectors recognize cell lines derived from multiple cancer histologies. Optimization of TCR expression and activity by modification of its nucleotide sequence allowed us to identify the optimal design for efficient expression and potent antigen-specific reactivity against SSX2. Production of a clinical-grade retroviral vector producer cell line was further pursued for its future application in adoptive immunotherapy clinical trials.

## Materials and Methods

### Cell lines and human PBLs

HLA-A*0201+/SSX2+ melanoma cell line 624, and non-HLA-A*0201 cell lines 888 and 938 were established from surgically resected metastatic melanoma tumors and maintained at the Surgery Branch, National Cancer Institute, National Institutes of Health (Bethesda, MD). The HLA-A*0201+/SSX2+ glioma cell line U251 was obtained from the Division of Cancer Treatment and Diagnosis Tumor Repository, National Cancer Institute, National Institutes of Health (Frederick, MD). Melanoma lines SKmel23 and SKmel37, and breast cancer cell line MCF7 were purchased from ATCC (Manassas, VA). Cos7-A*0201 and 293-A*0201 cells were retrovirally engineered to express HLA-A*0201 as described previously [19], [20]. Cos7-A*0201-SSX2 and 293-A*0201-SSX2 cells were transduced with a retroviral vector expressing the cDNA of SSX2. T2 is a lymphoblastoid cell line lacking TAP function, whose HLA class I proteins can be easily loaded with exogenous peptides. PG13 packaging clones were generated using the PG13 gibbon ape leukemia virus packaging cell line (ATCC CRL-10686), and the human ecotropic packaging cell line, Phoenix ECO (kindly provided by Dr. Hans-Peter Kiem, Fred Hutchinson Cancer Research Center, Seattle, WA). All cells were cultured in D10 medium consisting of high-glucose (4.5 g/L), Dulbecco's modified essential medium (DMEM, Invitrogen, Carlsbad, CA) supplemented with 10% fetal bovine serum (FBS, Hyclone, Logan, UT) and 6 mM glutamine (final concentration; Invitrogen, Carlsbad, CA). Cells were maintained at 37°C and 5% CO_2_.

Peripheral blood lymphocytes used in this study were obtained from melanoma patients treated in the Surgery Branch, National Cancer Institute, National Institutes of Health, on NCI Institutional Review Board-approved protocols. Patients provided written consent for procurement of tissues and use of these for research purposes, as detailed in protocol 03-C-0277. Human lymphocytes were maintained in AIM-V medium (Invitrogen, Carlsbad, CA) supplemented with 5% human AB serum (Valley Biomedical, Winchester, VA), 50 U/mL penicillin, 50 μg/mL streptomycin (Invitrogen), and 300 IU/mL IL-2 and maintained at 37°C with 5% CO2. SSX2 specific T cell clones were newly generated from tumor infiltrated lymph node-derived T cells of patients Lau 567 and Lau 672, similarly as described previously [18].

### Synthetic peptides

The SSX2_41-49_ peptide (KASEKIFYV) corresponding to amino acids 41 to 49 of SSX2, and homologous peptides derived from SSX1 (KYSEKISYV), SSX3 (KVSEKIVYV), SSX4 (KSSEKIVYV), SSX5 (KASEKIIYV), SSX6 (KFSEKISCV), SSX7 (KSLEKISYV), SSX8 (KYSEKISYV), SSX9 (KSSEKIIYV), SSX10 (KASEKILYV), IGSF22 (KESAKIFYD), ARHGAP1 (KFGQKIFYV), GPR82 (SCYEKIFYG), PHF8 (LKGEKIFYL), LIPM (TGQEKIYYV), SYT14 (IVGEKIFYL), TCOF1 (KASEKILQV), RBL2 (SPREKIFYY) and FRAS1 (SPREKIYYV) were synthesized by GenScript (Piscataway, NJ). Peptides were dissolved in DMSO and diluted in RPMI 1640 medium for loading of T2 cells. Binding affinity to HLA-A2*0201 was predicted for each peptide using NetMHC-3.0 [21]. Identification of peptides homologous to SSX2_41-49_ with a potential for cross-reactivity was performed by blast search (blastp algorithm).

### Construction of retroviral vectors for the expression of SSX2–specific HLA-A*0201–restricted TCRs

MSGV1-based retroviral vectors were constructed by overlapping PCR with the alpha- and beta-TCR chains arranged in the following order: TCR alpha-chain, linker peptide furin-SGSGP2A, TCR beta-chain, as previously described [22]. The cloned TCR inserts were verified by restriction enzyme profiling and direct DNA sequencing. The cDNA encoding for the codon-optimized version of the SSX2-specific TCR and a codon-optimized TCR with murine constant regions were synthesized by GenScript. The human-mouse hybrid version of TCR-5 was designed as previously described [23].

### T cell transduction

Retroviral supernatants were generated by transfecting each pMSGV1-SSX2-TCR plasmid along with a vector encoding RD114 envelope into 293-GP cells using the Lipofectamine 2000 reagent (Invitrogen) in Opti-MEM medium (Invitrogen) [22]. Viral supernatants were then loaded onto RetroNectin-coated (Takara Bio, Japan) non-tissue culture-treated six-well plates. PBLs were stimulated with OKT3 (50 ng/mL) and rhIL-2 (300 IU/mL) 48 h prior to transduction, and the transduction was carried out as described previously [24].

### Tetramer staining

HLA-A*0201–restricted SSX2–derived peptide SSX2_41-49_ (KASEKIFYV) were produced by the National Institutes of Health Tetramer Core Facility at Emory University (Atlanta, GA) using phycoerythrin (PE) as the fluorochrome. For evaluation of TCR transduction efficiency in T cell subsets, transduced T cells were stained with a FITC-labeled anti-human CD8 (BD Pharmingen, San Jose, CA) and with PE-labeled HLA-A*0201 tetramers. Cells were analyzed using a FACScan flow cytometer with CellQuest software (BD Biosciences) or FlowJo software (Tree Star, Ashland, OR).

### Cytokine release assay

TCR-transduced lymphocytes were tested for antigen-specific reactivity in cytokine release assays using peptide-loaded T2 cells or tumor cells. To that end, effector cells and target cells were cocultured at a 1∶1 ratio (1×10^5^ of each) in 200 uL of AIM-V medium in duplicate wells of a 96-well microplate. Culture supernatants were harvested 18–24 h after the initiation of coculture and assayed for interferon-γ (IFNg) by ELISA (Thermo Scientific).

### [^51^Cr] release assay

The ability of the transduced PBLs to lyse HLA-A*0201+ SSX2+ tumor cells was assessed using a standard [^51^Cr] release assay as described previously [25] Briefly, TCR-engineered PBLs were cultured with decreasing ratios of ^51^Cr-labeled target cells (E:T ratio) in AIM-V medium in 96-well U plates at 37°C for 4 h. Lysis was measured by [^51^Cr] release in the medium according to the formula: percent lysis  =  (sample release - minimum release)/(maximum release - minimum release) x 100%. Results expressed as average of duplicate samples.

### Generation of a PG13 packaging clone encoding an SSX2-specific TCR

A PG13 retroviral packaging cell clone was generated as described previously with the following changes [24]. Phoenix ECO cells were transfected with 9.5 μg of plasmid DNA (pMSGV1-SSX2.567.5-co) using the Lipofectamine 2000CD transfection reagent (LifeTechnologies, Carlsbad, CA). After 48 h supernatant was harvested and used to transduce retroviral packaging cell line, PG13. Non-tissue culture treated 6-well plates coated with 20 μg/mL RetroNectin as described by the manufacturer. Retroviral vector supernatant (4 mL) was added to each well followed by centrifugation (2000×g) at 32°C. After 2 h, supernatant was removed and 5×10^5^ PG13 cells were added to the well, centrifuged (1000×g) for 10 min at 32°C. Two rounds of transduction were performed and then PG13 packaging clones were generated by limiting dilution cloning. Due to lack of a selectable marker, high titer clones were identified by RNA dot blot as described previously [24], [26]. Retroviral vector from the 6 highest titer clones was generated as described. Briefly, 175 cm^2^ tissue culture flasks (Nunc, Cole-Parmer, Vernon Hills, IL) were seeded at 4×10^4^ cells/cm^2^, followed by a medium exchange (30 mL) on day 3. Supernatant was harvested 24 h later, aliquoted and stored at −80°C until further use. Supernatant from each clone was evaluated for the ability to efficiently transduce human PBL and elicit IFNg in a cytokine release assay. A high titer clone will be selected for production of a master cell bank and subsequent GMP retroviral vector supernatant.

### Generation of GMP retroviral vector supernatant

A total of 26 1700 cm^2^ expanded surface roller bottles were seeded on day 0 at a cell density of 4×10^4^ cells/cm^2^ in 200 mL of D10 medium. On day 3, the medium was exchanged and replaced with 120 mL D10 medium. Medium containing the retroviral vector was harvested daily with bottles being refed with 120 mL of medium. Glucose levels were monitored daily using Roche's Accu-check system (Roche, Basal, Switzerland). If glucose levels dropped below 2 g/L, the volume of the medium exchange was doubled to 240 mL/roller bottle for all subsequent harvests. All harvests were aliquoted and stored at −80°C until further use. An aliquot from each harvest was tested for transduction efficiency and cytokine release as described previously. All clinical products were subjected to an extensive biosafety testing program in accordance with current regulatory guidelines (US Food and Drug Administration, Center for Biologics Evaluation and Research; reference Points to Consider in the Production and Testing of New Drugs and Biologicals Produced by Recombinant DNA Technology, 1985; Points to Consider in the Characterization of Cell Lines Used to Produce Biologicals, 1993; Guidance for Industry: Guidance for Human Somatic Cell Therapy and Gene Therapy, 1998; Guidance for Industry, INDs – Approaches to Complying with CGMP During Phase I, 2006).

## Results

### Cloning of human SSX2-reactive TCRs

**Figure 1:**
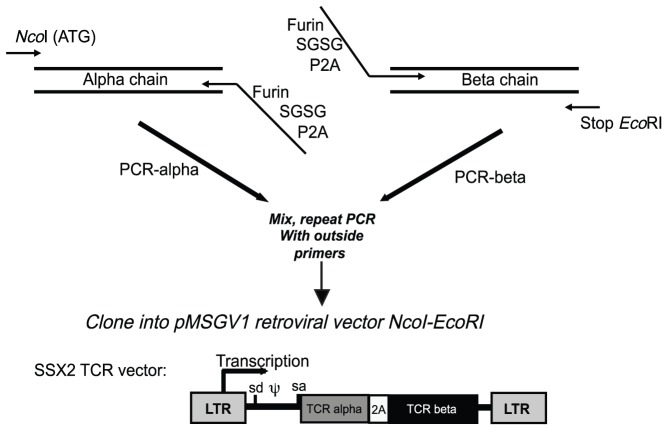
Generation of retroviral vectors for the expression of SSX2-specific TCRs. The coding region of each TCR alpha-chain was amplified by PCR using primers flanked by an NcoI restriction site in the 5′ end and an overhanging sequencing containing the element in the 3′ end. In parallel, each TCR beta-chain was amplified by PCR using a forward primer containing a 5′ overhang that overlaps with the 3′ overhang present in the primer used for the amplification of the alpha-chain. The reverse primer used for amplification of the beta-chain contained a stop codon and an *Eco*RI restriction site. In a second round of PCR, the products of the alpha- and beta-chain amplifications were pooled, and ligation of both cDNA fragments through the overlapping overhangs was achieved by PCR using external primers. The resulting PCR products were cloned in pMSGV1 vector for retrovirus production. LTR: long terminal repeat, sd: splice donor, sa: splice acceptor, ψ: retrovirus encapsidation signal.

The coding regions of TCR alpha- and beta- chains were cloned from previously described naturally occurring SSX2-reactive T cells from two melanoma patients [18]. These HLA-A2-restricted CD8 cells recognize an epitope spanning residues 41 to 49. cDNA was synthesized by 5′RACE using primers specific for the constant region of TCR genes and the products were sequenced, identifying seven different pairs of TRAV and TRBV genes (table 1). Expression cassettes containing the alpha- and beta-TCR chains separated by the 2A linker peptide [27], [28] were generated by overlapping PCR and cloned in to pMSGV1 for generation of retroviral expression vectors for clones 5, 8, 9 and 11 (figure 1A).

**Table 1:** TCRs isolated from SSX2-reactive lymphocyte clones.

Patient	T cell clone	TRAV	TRBV
Lau 567	5	14/DV4*01	15*02
	8	23/DV6*02	10-3*01
	10	17*01	15*02
Lau 672	7	12-2*02	7-9*01
	9	5*01	20-1*01
	11	12-1*01	2*01
	14	21*02	19*01

TRAV: TCR alpha-chain variable region.

TRBV: TCR beta-chain variable region.

### Expression of cloned TCRs in human lymphocytes

**Figure 2:**
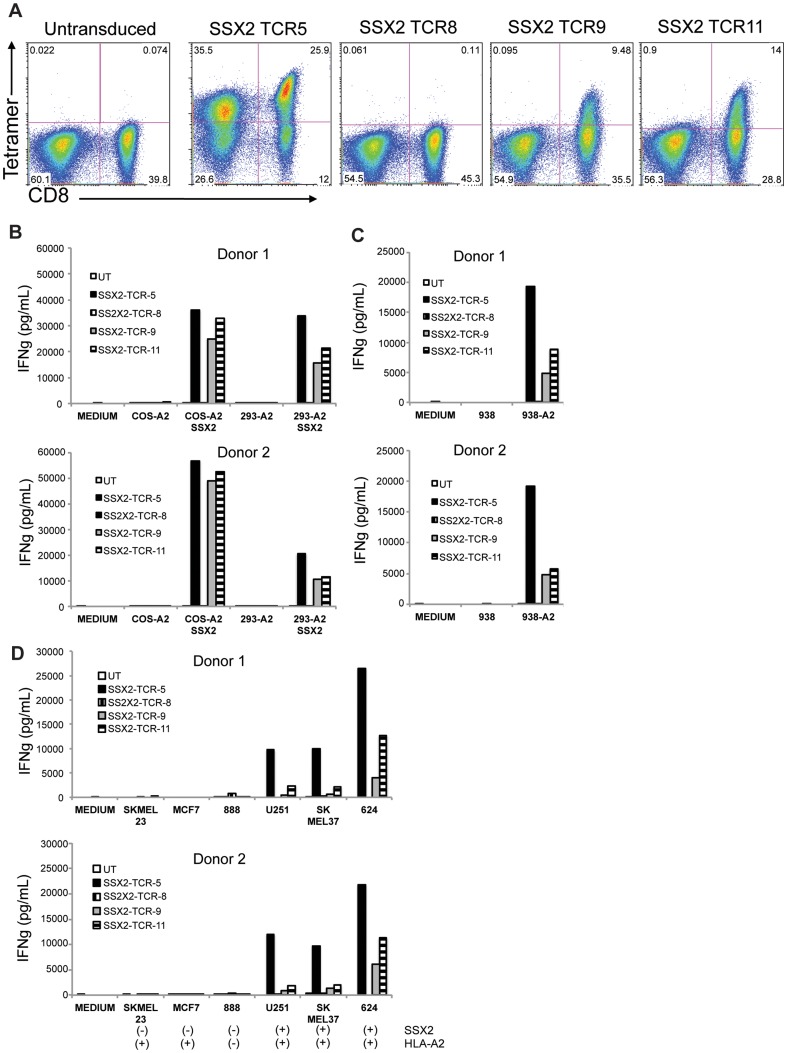
Expression and biological function of SSX2 TCR-5, -8, -9 and -11. A) Analysis of surface expression of SSX2_41-49_-specific TCRs in CD8 and CD4 T cell populations by flow cytometry. Human peripheral blood mononuclear cells were stimulated with OKT3 and transduced with the indicated retroviral expression vectors. One week later, cells were stained with anti-human CD3, CD8 antibodies and with a fluorescently labeled tetramer containing the SSX2_41-49_ peptide. Results from one representative donor of at least four independent experiments. Events gated on lymphoid, single, viable, CD3+ cells. B) – D) Concentration of IFNg in supernatants of TCR-transduced T cells cultured overnight with the indicated targets (1×10^5^ effectors vs 1×10^5^ targets). Results shown as average of duplicates for two representative donors out of four. UT: untranduced.

Retroviral vector supernatants were generated by transient transfection of 293GP and used for transduction of OKT3-stimulated human T cells. Expression and correct assembly of TCRs was evaluated by flow cytometry, using fluorescently labeled HLA-A2-SSX2_41-49_ tetramers. TCR5 was efficiently expressed in both CD8 and CD4 cells, TCR9 and TCR11 were expressed only in CD8 cells, while expression of TCR8 was not detected by this technique (figure 2 A).

### Reactivity of PBL transduced with human anti-SSX2 TCRs against SSX2-positive tumor cells

The correct processing and presentation of the SSX2_41-49_ epitope, and recognition by TCR-engineered PBL were tested in vitro by culturing the PBL with derivatives of Cos-7 and HEK293 cells that express HLA-A*0201 with or without concomitant expression of the antigen SSX2 (Cos-A2, Cos-A2 SSX2, 293-A2 and 293-A2 SSX2, respectively). As shown in figure 2B, T cells transduced with TCR-5, -9 and -11 secreted high levels of IFNg (in the order of 1×10^4^ pg/mL) when cocultured with SSX2-transfectants. TCR-8-transduced T cells secreted only background levels of IFNg, in line with the lack of tetramer binding shown in figure 2A. In order to verify the HLA-A*0201 restriction of these TCR, an SSX2-positive HLA-A*0201-negative melanoma cell line (938) or its derivative engineered to express HLA-A*0201 (938-A2) were used as targets for coculture experiments. As shown in figure 2C, lymphocytes expressing TCR-5, -9 and -11 secreted IFNg only when cultured in presence of 938-A2 cells. Recognition of 938-A2 endogenous SSX2 was stronger in TCR-5-transduced lymphocytes, as evidenced by a 2-fold higher IFNg secretion by those cells compared to that of T cells transduced with TCR-9 and -11 (figure 2C).

To test the ability of these TCRs to recognize the endogenous SSX2 expressed by tumor cells, we cultured PBLs (transduced with TCR-5, -8, -9, -11) with cell lines derived from melanoma (888, SKMEL-23, 624), glioma (U251), and breast cancer (MCF-7). As shown in figure 2D, TCR-5, -9 and -11 mediated IFNg release by transduced lymphocytes when cultured in presence of HLA-A*0201-positive SSX2-positive tumor cells from glioma and melanoma. Of note, TCR-5 mediated the strongest target recognition among the different TCRs test. Based on this, and on its efficient expression in both CD8 and CD4 T cells, we selected TCR-5 for further characterization.

### Cross-reactivity with other members of the SSX family and non-SSX proteins

**Figure 3:**
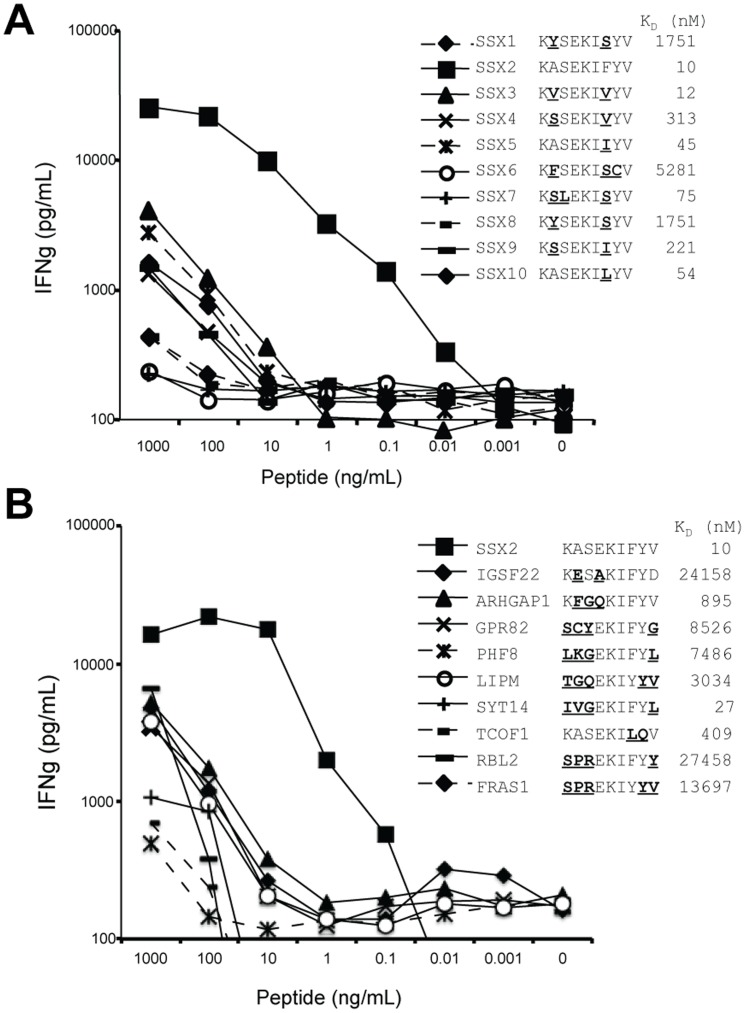
Analysis of recognition of other genes by TCR-5. Peripheral blood T cells expressing TCR-5 were cocultured overnight with T2 cells previously pulsed with the serial dilutions of the indicated peptides. Results of IFNg concentration in culture supernatants are expressed as average of duplicates in a representative experiment. Sequence alignment of the tested peptides is shown in the figure legend for A) SSX-family genes and B) non-SSX genes with overlapping sequences. IGSF22: immunoglobulin superfamily member 22, ARHGAP1: Rho GTPase-activating protein 1, GPR82: Probable G-protein coupled receptor 82, PHF8: histone lysine demethylase PHF8, LIPM: lipase member M, SYT14: synaptotagmin-14, TCOF1: treacle protein, RBL2: retinoblastoma-like protein 2, FRAS1: extracellular matrix protein FRAS1. Prediction of binding affinity to HLA-A2*0201 is shown for each peptide, expressed as dissociation constant (K_D_, nM).

The SSX family comprises 10 genes encoding proteins that are highly homologous to each other. In particular, the epitope of SSX2 targeted by TCR-5 lies within one of the regions of homology between members of the family. As shown in figure 3A, the amino acid sequence of SSX5 and SSX10 differ from SSX2_41-49_ in only one residue; SSX1, SSX3, SSX4, SSX8 and SSX9 differ from SSX2_41-49_ in two amino acids; and SSX6 and SSX7 differ from SSX2_41-49_ in three amino acids. In addition to SSX2, SSX3 and SSX5 were predicted to bind HLA-A2 molecules with high affinity. Peptides derived from SSX4, SSX7, SSX9 and SSX10 were predicted to bind to HLA-A2 with lower affinity. Reactivity of TCR-5 against each of these peptides was tested in coculture experiments using human lymphocytes expressing TCR-5 as effectors and peptide-pulsed T2 cells as targets, and secretion of IFNg was assessed as a marker of antigen recognition. While TCR-5-expressing cells secreted IFNg upon exposure to SSX2_41-49_ at low concentration of peptide (>0.01 ng/mL), they reacted to SSX3-, SSX4-, SSX5-, SSX9- and SSX10-derived peptides only when exposed to high concentrations of peptide (over 10 ng of peptide/mL, figure 3 A). This difference in reactivity of three to four orders of magnitude indicates that TCR-5 is relatively specific for SSX2_41-49_, and suggests that recognition of cells expressing homologous peptides derived from other SSX genes may be unlikely.

Nine additional protein-encoding genes were identified by blast search as partially homologous to the SSX2_41-49_ epitope: IGSF22, ARHGAP1, GPR82, PHF8, LIPM, SYT14, TCOF1, RBL2 and FRAS1. The overlapping peptides in two of these proteins, IGSF22 and TCOF1, differed in only two residues from the targeted SSX2 epitope. In addition, peptides derived from SYT14 and TCOF1 were predicted to be strong and weak HLA-A2 binders, respectively (figure 3B). We analyzed the recognition of these nonamer peptides by TCR-5 in coculture experiments using peptide-pulsed T2 cells. As depicted in figure 3B, IFNg release induced by SSX2_41-49_ peptide was two to four orders of magnitude superior to that induced by the homologous peptides, confirming the specificity of TCR-5.

### Codon optimization and murinization of TCR-5

**Figure 4:**
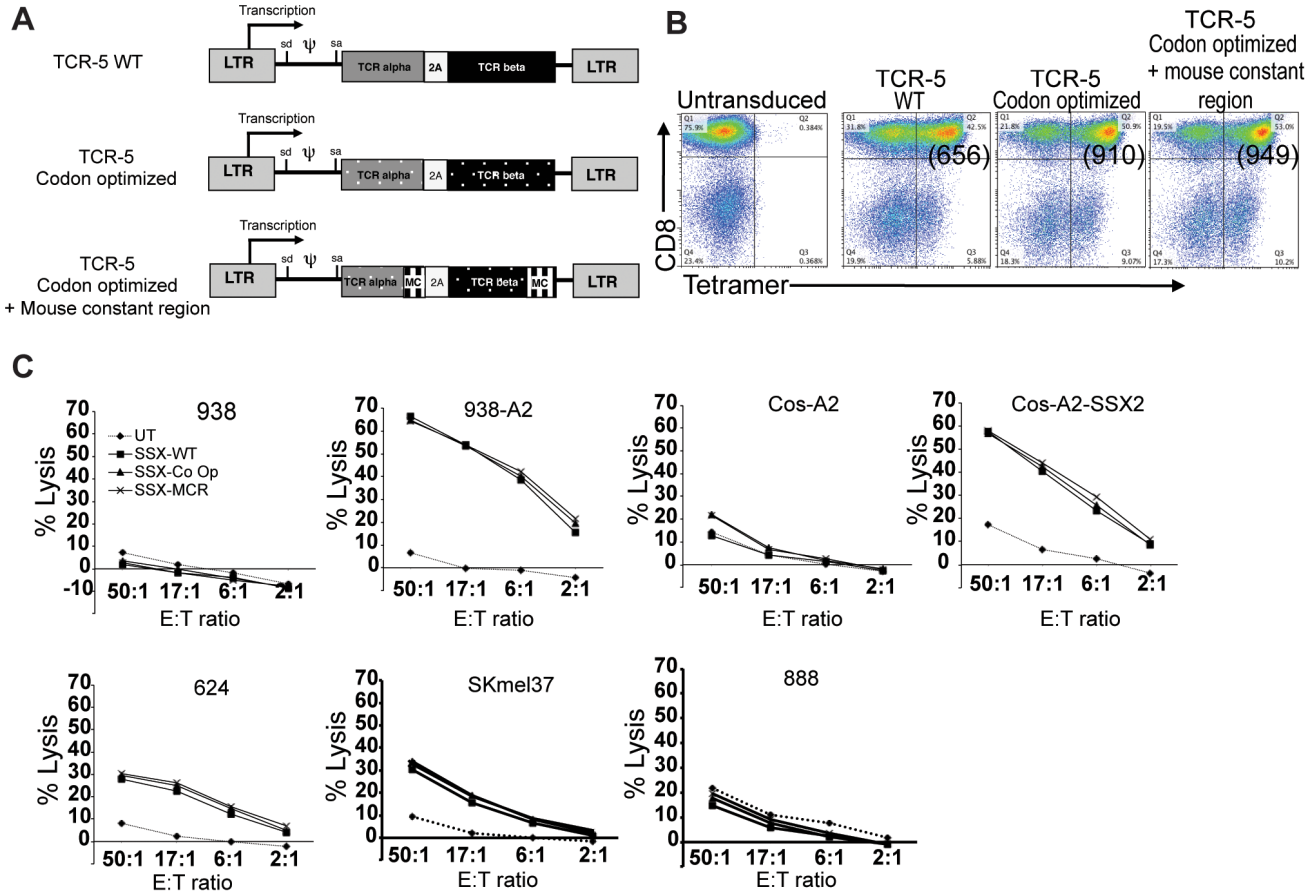
Codon optimization and replacement of TCR constant regions with murine sequences. A) Schematic representation of the three constructs generated for the expression of TCR-5 and derivatives. LTR: long terminal repeat, sd: splice donor, sa: splice acceptor, ψ: retrovirus encapsidation signal, MC: mouse TCR constant region, 2A: linker peptide. B) Analysis of expression of TCR-5 variants by tetramer staining. OKT3-stimulated lymphocytes were transduced twice with the corresponding TCR-expressing vector and stained with anti-CD3, anti-CD8 and SSX2_41-49_ tetramers one week after transduction. Representative results from three independent experiments. Values in parentheses represent the mean fluorescence intensity of tetramer staining within the CD8 T cell population. C) ^51^Cr-release assay for the evaluation of antigen-specific cytolysis induced by TCR-5-transduced lymphocytes after four-hour coculture with the indicated target cells. Percentage of lysis depicted for each target cell line at different effector:target ratios is the average of duplicates in a representative experiment of three independent experiments. UT: untransduced T cells used as negative control of unspecific lysis, WT: Wild-type TCR, Co Op: copon-optimized TCR, MCR: codon-optimized TCR with mouse constant region.

We next evaluated whether optimization of codon usage in the coding sequence of TCR-5 and/or the use of human-mouse hybrid TCRs could enhance TCR-5 expression and/or biological activity. cDNA encoding for the same amino acid sequence as TCR-5 but with a nucleotide sequence optimized for codon usage in humans was synthesized and cloned in pMSGV1 for generation of retroviral vectors. Similarly, a codon-optimized version of TCR-5 in which the TCR constant regions were replaced with the constant region of a mouse TCR [23] was synthesized and cloned in pMSGV1. The three versions of TCR-5 (WT, codon optimized, and codon optimized + mouse constant region, figure 4A) were next compared in their expression and ability to recognize antigen and mediate cell lysis.

After retroviral transduction of OKT3-stimulated human lymphocytes, tetramer staining was used to evaluate the expression of the three constructs. As shown in figure 4 B, codon optimization increased the density of membrane expression of TCR in CD8 T cells as evidenced by an increased mean fluorescence intensity (MFI) of SSX2_41-49_ tetramer staining in cells transduced with the codon-optimized TCR-5 (910 fluorescence units), compared to those transduced with the wild-type version (656 fluorescence units). However, use of a murine constant region in addition of codon optimization only minimally increased the tetramer binding by the TCR (949 fluorescence units). The biological activity of the TCRs was tested in coculture experiments where the TCR-transduced lymphocytes were exposed to multiple HLA-A*0201-positive target cells that were positive for SSX2 (Cos-A2-SSX2, 293-A2-SSX2, K562-A2, 624, 938-A2 and U251). The concentration of IFNg in the supernatants was measured by ELISA after an overnight coculture and the results are shown in table 2. Lymphocytes transduced with the codon-optimized version of TCR-5 secreted, on average, 30% more IFNg than those transduced with the wild-type TCR. The presence of a mouse TCR constant region also increased IFNg secretion compared to the wild-type TCR, but this modification did not increase the IFNg secretion of the codon-optimized variant (table 2). The three constructs displayed similar properties in terms of induction of antigen-specific proliferation of T cells in thymidine uptake assays (figure S1A), T cell activation upon antigen recognition (evidenced by up-regulation of the activation marker CD137, figure S1B) and interleukin-2 (IL-2) production (figure S1C).

**Table 2:** IFNg secretion (pg/mL) by TCR-5 transduced lymphocytes upon coculture with indicated targets.

Target cell line	Histology				TCR-5									
			UT			WT			Codon Optimized				Codon optimized + mouse constant region	
Cos-A2-SSX2	Kidney (Monkey)	615			52577			64142				56893		
293-A2-SSX2	Kidney	515			29804			37522				37258		
K562-A2	Erythroleukemia	1830			12542			21325				17437		
SKmel37	Melanoma	96			6635			8869				10401		
624	Melanoma	453			27344			37547				46999		
938-A2	Melanoma	626			37032			46304				51092		
U251	Glioma	372			16653			19223				19027		
SKOV3	Ovarian cancer	877			2414			2527				2221		

### Induction of cell lysis of tumor cells expressing SSX2

The ability of each variant of TCR-5 to induce antigen-specific cell lysis of tumor targets by human lymphocytes was determined using a chromium release assay. Chromium (^51^Cr)-labeled 938, 938-A2, Cos-A2, Cos-A2-SSX2, 624, SKmel37 and 888 cells were cocultured with human peripheral blood T cells transduced the wild-type TCR-5 or its codon-optimized or codon-optimized murinized variants, at different effector:target ratios. Untransduced T cells were used as negative control. As depicted in figure 4 C, lymphocytes transduced with either of the SSX2_41-49_-specific TCRs induced potent cytolytic effect on 938-A2 cells but not on 938 cells, indicating that lysis is HLA-A*0201-restricted. In addition, Cos-A2-SSX2 cells were lysed by TCR-5-expressing cells, but not Cos-A2, which lack expression of SSX2, indicating that this effect is antigen-specific. Similarly, 624 and SKmel37 cells, which are HLA-A*0201 positive and naturally express SSX2, were lysed by lymphocytes transduced with either TCR-5 variant, whereas HLA-A*0201-negative 888 cells were not. Together, these results confirm that TCR-5-derived constructs are biologically functional in human peripheral blood T cells, and that they can be used to redirect their antigen specificity to SSX2, in an HLA-A*0201-specific fashion. Neither codon optimization or murinization of TCRs had an effect on TCR-5-mediated lysis.

### Development and testing of clinical grade retroviral vector supernatants

**Figure 5:**
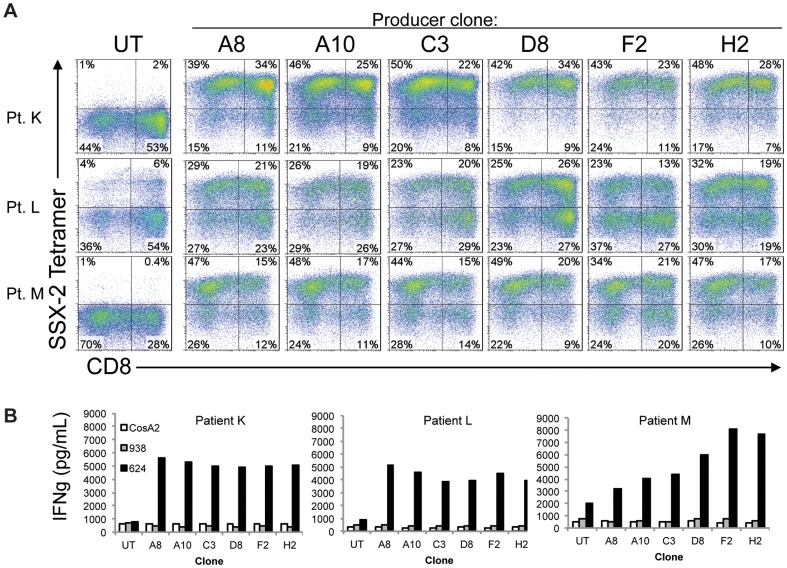
Analysis of expression and biological function of codon-optimized TCR-5 in human lymphocytes transduced with clinical grade retroviral vector supernatants. A) Flow cytometry analysis of tetramer staining of T cells transduced with vector supernatants produced by stable packaging line clones A8, A10, C3, D8, F2 and H2. Clones were previously selected as those displaying the highest expression of viral RNA using dot plot analysis. Results of tetramer and CD8 staining of T cells are shown for three patients. B) IFNg ELISA of supernatants from cocultures of T cells transduced with vectors derived from clones A8, A10, C3, D8, F2 and H2. HLA-A*0201+ SSX2+ melanoma cells 624, HLA-A*0201- SSX2+ melanoma cells 938 and HLA-A*0201+ SSX2- cells CosA2 were used as targets.

Due to the tumor-selective pattern of expression of SSX2, and the potent yet selective antigen-recognition properties of TCR-5, we decided to produce and test clinical-grade retroviral vectors suitable for the expression of TCR-5 in peripheral blood T cells of cancer patients. Stable packaging lines were established by transduction of PG13 cells with a retroviral vector encoding the codon-optimized version of TCR-5. After two transductions, PG13 cells were cloned by limiting dilution and clones were tested for viral RNA expression using dot–plot (not shown). The six clones expressing the highest levels of vector RNA (A8, A10, C3, D8, F2 and H2) were amplified and the supernatants were tested for their ability to induce TCR expression in OKT3-stimulated PBL from three different donors. Tetramer staining of transduced T cells revealed that supernatants of the six clones mediated efficient transduction of lymphocytes (figure 5 A). The average percentage of transduction of clones A8 A10, C3, D8, F2 and H2 (taking CD8 and CD4 T cells together) were 61.7%, 60.3%, 58.0%, 65.3%, 52.3% and 63.7%, respectively. In agreement with their similar transduction efficiency, supernatants of all clones induced reactivity against HLA-A*0201-positive SSX2-expressing 624 cells when tested in coculture assays (figure 5 B). IFNg release after overnight coculture of 1×10^5^ lymphocytes with 1×10^5^ 624 cells was (5023+/- 295) pg/mL, whereas transduced lymphocytes released only background levels of IFNg when exposed to HLA-A*0201-negative 938 cells or SSX2-negative CosA2 cells.

Together, these results document the development of a clinical grade reagent for TCR-based adoptive immunotherapy of human malignancies, targeting the cancer-testis antigen SSX2 using a codon-optimized TCR derived from a melanoma patient.

## Discussion

Cancer-testis antigens have been proposed as ideal targets for adoptive immunotherapy of cancer due to their over expression in tumor cells of multiple histologies and restricted expression in normal tissues [11]. Although SSX2 is expressed in male germ cells, the lack of expression of MHC molecules by these cells prevents antigen presentation on the cell surface. We focused on one specific cancer-testis antigen, synovial sarcoma X breakpoint (SSX)2, a gene initially described as part of chromosomal rearrangements commonly found in synovial sarcoma cells: SYT-SSX2 [29] and SS18-SSX2 [30] translocations. The gene products resulting from these translocations have been shown to act as epigenetic modulators and to regulate cell growth, proliferation, cell death, potentially contributing to oncogenesis [30]. The physiological function of wild-type SSX2, however, is largely unknown.

SSX2 expression has been documented on prostate cancer [31], [32] and in multiple myeloma [33], [34] in addition to melanoma [17]. Initial studies by Tureci *et al* showed SSX2 mRNA was expressed in 50% of melanomas, 30% of hepatocarcinomas, 25% of colon cancers, and 20% of breast carcinomas [17]. Dubovsky and McNeel reported that SSX2 mRNA was expressed in testis, but undetectable in liver, lung, colon, bladder, heart, brain, kidney, placenta, skeletal muscle, spleen, thymus, or prostate, by RT-PCR [10]. Similarly, no expression was detected in five normal prostate epithelial cell lines, but SSX2 mRNA was expressed by LAPC4 and MDA-PCa-2b prostate cancer cell lines. Interestingly, SSX2 mRNA expression was increased by treatment with demethylating agent 5-aza-2′deoxycytidine (AZA) in the prostate cancer cell lines LNCAP and DU145 but not in the normal prostate epithelial cell line RWPE-1 [10]. More recently, Smith *et al* performed an immunohistochemical analysis of prostate cancer resections using an antibody that recognizes both SSX2 and SSX3. They found that 25% of metastatic lesions were positive for SSX2/3, but normal prostate and primary lesions were negative [31], [32].

Dos Santos *et al* detected SSX2 mRNA expression in nine of eighteen melanoma cell lines by RT-PCR, and positive staining in 34 of 101 melanoma lesions using a monoclonal antibody that recognizes SSX2, SSX3 and SSX4 proteins [35]. Taylor *et al*. reported the detection of SSX2 mRNA in 24% of bone marrow samples of multiple myeloma patients (total: 114) [36], while Atanackovic *et al*. detected SSX2 in 12%–16% of bone marrow samples with significant plasma cell infiltration, but not in bone marrows of healthy donors [33], [37]. This expression across different cancer histologies makes SSX2 an attractive target that may allow for treatment of patients with different diagnoses using a single clinical grade reagent. Curative treatment options for patients with metastatic androgen-independent prostate cancer or multiple myeloma are currently scarce, highlighting the need for novel treatment modalities [38], [39]. In this context, adoptive immunotherapy targeting SSX2 might have a substantial impact in the clinical management of such diseases.

The SSX2 protein can induce spontaneous immune responses in cancer patients. Evidence of SSX2 immunogenicity includes the detection of SSX2-specific antibodies in the serum of patients with melanoma, colon cancer and breast cancer [40] and the presence of CD8 T cells with specificity for SSX2 in tumor-infiltrated lymph nodes of patients with melanoma and hepatocarcinoma [41], [42]. These CD8 T cells recognized an epitope spanning residues 41–49 of SSX2, in a HLA-A*0201-restricted fashion and were found to be selectively expanded in patients with SSX2-expressing cancers [18], [42]. In the present study we sought to isolate the SSX2_41-49_-specific TCRs expressed by the CD8 T cells of those patients, and to develop and characterize retroviral vectors for the expression of those TCRs in human peripheral blood T cells. This strategy would allow us to by-pass the induction of a natural immune response against SSX2 by generating large numbers of T cells with specificity for this antigen, that could potentially be used for adoptive cell transfer studies.

A total of seven pairs of TCR alpha- and beta-chains were identified from melanoma patients Lau 567 and Lau 672. The cDNA encoding for four of these TCRs were cloned in retroviral expression vectors and tested for expression in transduced human T cells and for biological activity. While the TCRs derived from clone 5 of patient Lau 567 and clones 9 and 11 of patient Lau 672 were efficiently expressed in CD8 T cells upon retroviral transduction, the TCR isolated from clone 5 (TCR-5) was expressed, and functionally active, in both CD8 and CD4 T cell populations, despite having been derived from a CD8 T cell clone. This characteristic may allow for the generation of more potent immune responses against SSX2 by activating both the cytolytic function of CD8 T cells and the cytokine-secreting ability of CD4 T cells [43], [44].

Smith and collaborators isolated T cells that recognized a SSX2 epitope spanning residues 103–111, from a prostate cancer patient. These T cells, however, also recognized the equivalent epitope in SSX-3, -5 and -9 [31], [32]. The remarkable specificity of TCR-5, even among highly homologous peptides derived from other SSX family members, has important implications in terms of safety. This is relevant considering that a TCR directed against another cancer-testis antigen, MAGE-A3, previously generated in our laboratory [45] induced tumor regression but also severe neurotoxicity in a Phase I clinical trial of adoptive immunotherapy. These adverse events were likely due to cross-reactivity against an epitope present in MAGE-A12, which has previously unreported expression in a small subset of neurons [46]. This epitope of MAGE-A12 differed from the MAGE-A3 epitope targeted by the TCR in only one amino acid, but this difference was associated with a significant increase in the affinity of binding to HLA-A2. In fact, the MAGE-A12-derived peptide was recognized by the MAGE-A3 TCR with higher avidity than the MAGE-A3 peptide [46]. Such cross-reactivity against other members of the SSX family is unlikely to occur *in vivo* with SSX2 TCR-5, because the binding affinities of this TCR for the related peptides was at least three orders of magnitude lower than the affinity for SSX2_41-49_, in *in vitro* experiments.

Several modifications can be introduced in the TCRs in order to increase their affinity or avidity for their cognate antigens. In the present study we analyzed two ways of potentially increasing the expression of functional TCRs in the surface of transduced T cells: codon optimization and codon optimization plus replacement of the constant region of the TCR chains with the constant regions of murine origin. Optimization of codon usage for expression in human cells was found to increase both the surface expression of the SSX2-specific TCRs in transduced T cells and the reactivity of these T cells against SSX2-expressing targets. We had previously shown that replacement of the TCR constant region by constant regions of murine origin could increase the TCR expression and prevent mispairing of the inserted TCR chains with the endogenous TCR molecules, potentially preventing the generation of toxic neospecificities [23]. The theoretical possibility of off-target toxicity resulting from those neospecificities has prompted researchers to develop multiple strategies to prevent mispairing, including transcriptional silencing [47] or genetic ablation [48] of endogenous TCR expression. In the case of SSX2 TCR-5, no additional increase in expression, tetramer binding or activity was provided by usage of murine constant regions, suggesting that mispairing is negligible if at all existing. The three versions of TCR-5 displayed similar properties in terms of proliferation in response to antigen stimulation and cytolytic capabilities. Therefore, we selected the codon-optimized version of TCR-5 for further development, in order to prevent potential antibody responses against murine sequences that may inhibit the activity of TCR gene-modified cells [49]. Amino acid modifications can also be introduced in the complementarity determining regions (CDR) of TCRs to enhance their affinity for a given target [45], [50]. Such modifications, however, can induce the recognition of non-related proteins [50], making it complicated to predict potential cross-reacting peptides. A more extensive preclinical evaluation of tissue recognition would therefore be required involving, for instance, coculture experiments with multiple cell types derived from induced pluripotent stem cells [50]. One advantage of non-modified human TCRs such as TCR-5 is that they have undergone thymic negative selection of self-reactive clones, and therefore are less likely to recognize vital self-tissues.

An important feature of cancer-testis antigens is that their expression can be induced by epigenetic modification of the tumor cells as a consequence of treatment with pharmacological agents. For instance, treatment with demethylating agents such as AZA and/or histone deacetylase inhibitors such as depsipeptide have been shown to increase the levels of expression of NY-ESO-1 in melanoma cells, leading to increased recognition by T cells transduced with anti-NY-ESO-1 and anti-MAGE-A3 TCRs [22], [41]. Therefore, the development and characterization of retroviral vectors expressing SSX2 TCR-5 as a clinical grade reagent open a wide array of possibilities in the design of adoptive immunotherapy of cancer. These include not only the utilization of autologous T cells transduced with SSX2 TCR-5 as a single agent, but also combination therapies consisting of sequential administration of demethylating agents and/or deacetylase inhibitors with T cells targeting one or more cancer-testis antigens [51]. A careful validation of the optimal dosage and scheduling would be necessary to guarantee the induction of the expected synergism between the two parts of the treatment.

## Supporting Information

Figure S1 **Antigen-driven proliferation and activation of human T cells expressing either wild-type, codon-optimized or codon-optimized ‘murinized’ TCR-5.** A) Proliferation of TCR-5-transduced lymphocytes after coculture with CosA2-SSX2 cells during three days. Values represent incorporated [3H]thymidine as average counts of triplicate wells. Co op: codon optimized; MCR: codon-optimized plus mouse constant region. B) Flow cytometry analysis of CD137 (4-1BB) expression in TCR-5-transduced T cells upon coculture with HLA-A*0201+ SSX2+ or HLA-A*0201+ SSX2- cells (Cos-A2-SSX2 and Cos-A2, respectively). Staining with anti-CD8 and anti CD137 antibodies was performed after an overnight coculture. C) Flow cytometry analysis of IFNg and IL-2 expression in TCR-5-transduced T cells upon coculture with HLA-A*0201- SSX2+ or HLA-A*0201+ SSX2+ cell lines (938 and 938-A2, respectively). Gated on CD3+ cells.
(TIF)

Methods S1 (DOCX)
