# Supplementary figures and images for: Development of a T Cell Receptor Targeting an HLA-A*0201 Restricted Epitope from the Cancer-Testis Antigen SSX2 for Adoptive Immunotherapy of Cancer
Source: PLoS One. 2014 Mar 28;9(3):e93321. doi: 10.1371/journal.pone.0093321 (PMC3969312; doi:10.1371/journal.pone.0093321)

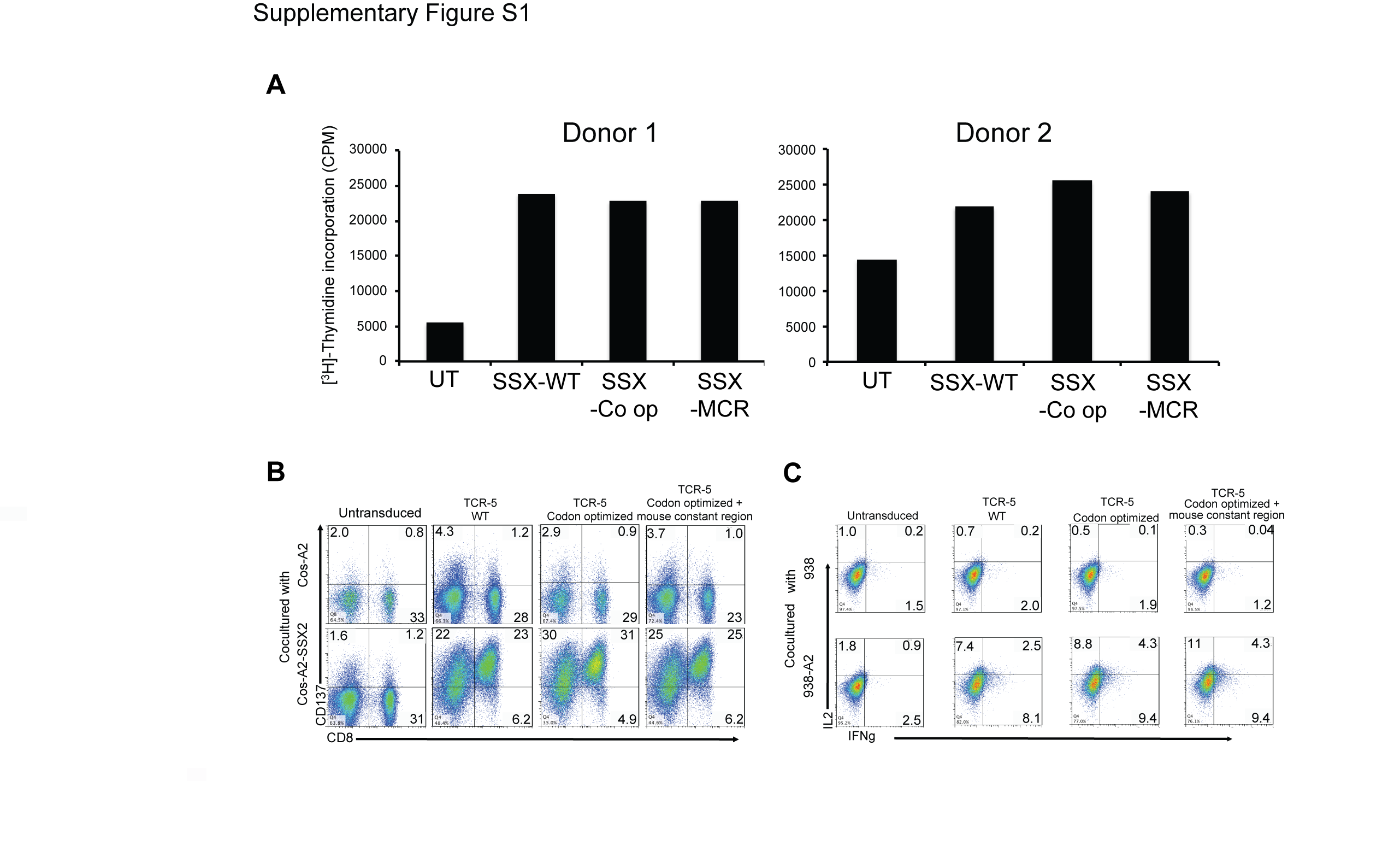

Supplement: Figure S1 — Antigen-driven proliferation and activation of human T cells expressing either wild-type, codon-optimized or codon-optimized ‘murinized’ TCR-5. A) Proliferation of TCR-5-transduced lymphocytes after coculture with CosA2-SSX2 cells during three days. Values represent incorporated [3H]thymidine as average counts of triplicate wells. Co op: codon optimized; MCR: codon-optimized plus mouse constant region. B) Flow cytometry analysis of CD137 (4-1BB) expression in TCR-5-transduced T cells upon coculture with HLA-A*0201+ SSX2+ or HLA-A*0201+ SSX2- cells (Cos-A2-SSX2 and Cos-A2, respectively). Staining with anti-CD8 and anti CD137 antibodies was performed after an overnight coculture. C) Flow cytometry analysis of IFNg and IL-2 expression in TCR-5-transduced T cells upon coculture with HLA-A*0201- SSX2+ or HLA-A*0201+ SSX2+ cell lines (938 and 938-A2, respectively). Gated on CD3+ cells. (TIF) [file pone.0093321.s001.tif]
