# Supplementary material for: Development of a T Cell Receptor Targeting an HLA-A*0201 Restricted Epitope from the Cancer-Testis Antigen SSX2 for Adoptive Immunotherapy of Cancer
Source: PLoS One. 2014 Mar 28;9(3):e93321. doi: 10.1371/journal.pone.0093321 (PMC3969312; doi:10.1371/journal.pone.0093321)
Supplement: Methods S1 — (DOCX) [file pone.0093321.s002.docx]

**SUPPLEMENTARY METHODS**

**[^3^H]-Thymidine incorporation assay.**

TCR-transduced T cells (1 x 10^5^) were cocultured with equal number of irradiated (18,000 rad, cesium source) Cos-A2-SSX2 target cells in AIM-V medium in a final volume of 0.2 mL in triplicate wells of a 96-well U-bottom microplate. Cells were maintained in culture for 3 days and pulsed with 1 mCi [^3^H]thymidine (DuPont, New England Nuclear, Shelton, CT) per well. After an additional 18 h culture, the cells were harvested onto a glass fiber filter (Wallac Oy, Turku, Finland), and radionucleotide incorporation (in count per minutes, cpm) was measured using a Perkin-Elmer Microbeta Trilux counter (Shelton, CT).

**CD137 expression and intracellular cytokine staining.**

APC-H7–conjugated anti-CD3 (SK7), APC-conjugated anti–CD137/4-1BB (4B4-1), PE-Cy7-conjugated anti-CD8 (SK1), PE-conjugated anti-IFNg (XMG1.2) and FITC-conjugated anti-IL2 (5344.111) antibodies were purchased from BD Biosciences (San Diego, CA). For cytokine intracellular stainings, monensin treatment was started one hour after the cocultures were plated and staining was performed four hours later, unless otherwise stated. BD Cytofix/Cytoperm with GolgiPlug was used as instructed by manufacturer (BD Biosciences).
